# Advantages and drawbacks of life cycle assessment application to the pharmaceuticals: a short critical literature review

**DOI:** 10.1007/s11356-024-33964-w

**Published:** 2024-06-19

**Authors:** Marco Satta, Fabrizio Passarini, Daniele Cespi, Luca Ciacci

**Affiliations:** 1 Department of Industrial Chemistry “Toso Montanari”, University of Bologna, Via Piero Gobetti 85, 40136 Bologna, Italy; 2 Interdepartmental Centre of Industrial Research “Renewable Resources, Environment, Sea and Energy”, University of Bologna, Via Angherà 22, 47922 Rimini, Italy

**Keywords:** Life cycle assessment, pharmaceuticals, API

## Abstract

Pharmaceuticals are among the most challenging products to assess by life cycle assessment (LCA). The main drawback highlighted by LCA practitioners is the lack of inventory data, both regarding the synthesis of active pharmaceutical ingredient (API) precursors (upstream) and the details concerning the downstream phases (use and end of life). A short critical review of pharma-LCAs found in the literature is here proposed, with discussion of several tools and models used to predict the environmental impacts derived from the life cycle of pharmaceuticals, emphasizing current strengths and weaknesses, and exploring the possibilities for improvements. The case of antibiotics is selected as a representative class of pharmaceuticals, due to their massive use worldwide and the growing related issue of antimicrobial resistance enrichment, which is generally not included in most of LCAs. Also, we comment on drafting product category rules (PCRs) in the relevant field to develop standard methodologies and enhance the comparability of the studies, ultimately advocating collaboration with companies and improving inventory data quality and availability for the whole value chain of products.

## Introduction

Over the last 40 years, the European Union (EU) has been strongly committed to implement the concept of sustainability in the chemical industry, reducing greenhouse gas (GHG) emissions in the sector by 54% since 1990, equal to 154 Mt of CO_2_eq. In addition, from 2007 the total amount of waste was reduced of nearly one-third, the accidental pollutant releases were dropped by at least 40% and the emission of water pollutants was nearly halved (CEFIC 2023). The concept of green chemistry (GC) was first introduced in 1991 by Paul Anastas and Roger Garrett and was later emphasized by the establishment of the 12 GC principles by Anastas and Warner (1998), driving the research in the field toward alternative, more efficient technologies with the aim to minimize the hazards derived from chemical processes and related wastes generated. In 2006 the REACH legislation was introduced under the EU’s regulation, to improve knowledge on the possible dangers and risks that could arise from existing and new chemical substances, maintaining the competitiveness and innovation capacity of the EU chemical industry (ECHA 2023). However, a review recently published by the European Court of Auditors addressed the fact that the amount of hazardous wastes generated by EU countries continued to increase in the years from 2004 to 2020 (ECA 2023).

In this context, the pharmaceutical industry plays a critical role mainly for two reasons. Firstly, it produces specific drugs, usually through complex synthetic pathways with a depletion of resources and generation of wastes that are significantly high compared to the low amounts of final product obtained (Health Care Without Harm 2019). This relates with the upstream phase, i.e., the early stages of the life cycle of pharmaceuticals, typically including the extraction of raw materials and the production of chemical precursors that are later employed in the core phase for final product manufacturing. Secondly, the active pharmaceutical ingredients (APIs) synthesized could severely affect the ecosystem if released into the environment, being specifically designed to be biologically active (Moermond et al. 2022). This factor instead deals with the downstream phase of the life cycle, which involves the activities related to products use and end-of-life (EoL) considerations. The raising awareness of these issues in our society is driving the research in the field toward a comprehensive evaluation of the impacts of products, in order to formulate plans and actions for a sustainable fine chemicals and pharmaceutical industry. To this aim, life cycle assessment (LCA) (ISO2006a; ISO 2006b) is recognized as a preferred methodology to evaluate the potential environmental impacts related to the value chain of products “from cradle to grave” (Anastas and Lankey 2000), and its implementation by pharmaceutical companies is constantly increasing, following a general trend in the manufacturing sector.

However, an examination of the existing LCA studies highlighted some important shortcomings in the methodologies and in the approach followed, deriving from the complex value chain of pharmaceuticals, which involves a broad range of factors beyond direct companies’ burdens. A great limitation that has been recognized is the lack of accurate, compliant, and consistent inventory data regarding product life cycle, something that is strongly connected to both the upstream and downstream phases and poses a serious limit to achieving the goal of a more sustainable production. Thus, a short review of the application of LCA to the pharma sector is presented, focusing on the current methodologies and on the main challenges that scientists are facing in this topic, by also highlighting the need for the development of common product category rules (PCRs) to be widely accepted. The advantages and drawbacks of LCA application to pharmaceuticals are discussed in detail in the next section, and the key aspects are summarized and ranked in Table 1. The discussion of these aspects is divided into two separate sections due to the different natures of the factors involved in the two phases, with particular emphasis placed on antibiotics as a case study, among the APIs more used in medicine (Cook and Wright 2022) and whose harmful effect on the environment is largely debated in literature (Kümmerer 2009; Polianciuc et al. 2020). Moreover, their use gives rise to a serious concern globally for human health due to the occurrence of antimicrobial resistance (AMR) (WHO 2020, an aspect that is still missing in pharma-LCA since a model to quantify the impacts of AMR in LCA studies of products associated with the use of antibiotics does not exist yet. Thus, the last part discusses two possible approaches to include AMR enrichment in pharma-LCA, as proposed by Nyberg et al. (2021). The two approaches are presented in detail and commented on, highlighting the strengths and weaknesses of each approach and the possibilities for application and improvement.

**Table 1:** Short summary of the main advantages and disadvantages of LCA application to pharmaceuticals

Advantages	Drawbacks
Formulation of plans and actions for a sustainable pharmaceutical and fine chemicals industry. ★★★	Lack of accurate LCI data, especially in large scale, due to confidentiality issues. ★★★
Identification of the most impactful stages of the supply chain of pharmaceuticals. ★★★	Value chain complexity, due to the great variability of regulations and production processes between different regions. ★★★
Preservation of human healthcare without affecting the environment. ★★	Lack of general and universally accepted rules to perform LCA studies of pharmaceuticals. ★★★
Improvement of quality communication between companies (B2B) and toward consumers (B2C). ★★	Insufficient quality and quantity of data regarding APIs emission into the environment. ★★
Comparative LCA studies for the development of sustainable synthetic routes for APIs. ★★	Lack of any model to implement AMR into LCA studies of products related with the use of antibiotics. ★★
Conscious choice between equivalents medications for the treatment of a disease, driving the purchase towards more sustainable options. ★	Disagreements in the healthcare sector on the use of the ATC classification system. ★
Overcoming of limitations of green metrics. ★	Definition of burdens and/or system boundaries not straightforward. ★

Stars represent the rating of importance, from the highest (★★★) to the lowest (★)

## Life cycle of pharmaceuticals: background

**Fig. 1:**
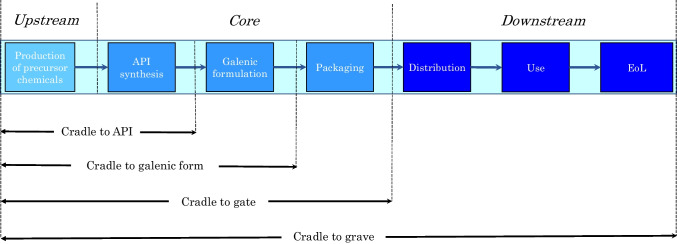
Generic life cycle of a pharmaceutical product, adapted from Siegert et al. (2019a)

Pharmaceutical industries generate more waste per unit product compared to any other chemical sector such as oil refining, bulk, and fine chemical industries (Phan et al. 2015; Sheldon 2007). Companies shall employ a holistic approach, implementing a life cycle perspective in their procedures to consider the direct/indirect emissions and resource consumption, as well as the production of hazardous/non-hazardous waste among the whole supply chain from raw material extraction (cradle) and production of precursors to manufacturing, use, and EoL phase (grave) (Jiménez-González and Overcash 2014). In a general LCA three main bulk phases can be identified, the upstream, core, and downstream, although a clear distinction is not always straightforward. Usually, the upstream phase includes the extraction and further processing of the starting resources (e.g., from fossil or bio sources), their supply to the facilities (e.g., inbound transportation), and the synthesis of precursors. The core phase generally covers the stages in which the synthesis and isolation of the API of interest, the galenic formulation with the incorporation of additives, and the final packaging of the product may occur. Finally, the downstream phase consists of the distribution, use, and EoL of the final product, including the analysis of the possible impacts derived from a release into the environment (Siegert et al. 2019a). With this schematization in mind (Fig. 1), some shortcomings in the LCAs of pharmaceuticals may often affect the modeling of chemical precursors production (upstream) and the EoL phase (downstream), as we discuss below.

### Upstream processing

The first issue relates to the definition of the system boundaries of the upstream phase since pharmaceutical companies often do not directly produce the chemical precursors but purchase them from trade partners. In these cases, the emissions and environmental impacts associated with the raw materials supply are seldom considered, resulting in an underestimation of environmental burdens of the final product (Milanesi et al. 2020; Jiménez-González et al. 2004a). Therefore, it becomes important for pharmaceutical industries to broaden their system boundaries incorporating the first stages of the supply chain of products in their LCAs. To minimize the impacts derived from the synthesis of chemical precursors, the application of the GC principles in the synthesis of fine chemicals has become essential. The use of common green metrics such as E-factor (Sheldon 1992; Sheldon 1997), atom economy (Trost 1991), and process mass intensity (Curzons et al. 2001) could help in assessing the environmental drawbacks associated with organic processes and supporting the selection of the most sustainable production routes (Sheldon 2018; Anastas et al. 2018). The American Chemical Society’s Green Chemistry Institute established in 2005 the ACS GCI Pharmaceutical Roundtable with the aim to stimulate the integration of GC in the pharmaceutical industry, defining PMI as the key parameter to express sustainability (Jiménez-González et al. 2011a; Jiménez-González et al. 2013). However, the potential impacts derived from toxicity and safety of the products and wastes are not considered by green metrics (Rose et al. 2022; Jiménez-González et al. 2011b); therefore, it is strongly recommended to conduct a comprehensive LCA to analyze and quantify the potential environmental impacts of chemical processes and products (Cespi et al. 2015; Cespi et al. 2020).

The use of huge amounts of volatile organic solvents plays an important role in the environmental impacts of chemical processes (Raymond et al. 2010), and much effort goes into replacing them with greener options (Sheldon 2005; Byrne et al. 2016; Clarke et al. 2018). Scientists from GlaxoSmithKline plc (GSK) developed in 1999 an internal guideline for the selection of the best solvent for each reaction (Curzons et al. 1999), further extended to reagents only in 2013 (Adams et al. 2013). As reported in the literature (Alder et al. 2016), this procedure was later integrated with the usage of a simplified LCA tool to facilitate and support the users to choose sustainable alternatives (Jiménez-González et al. 2004a; Curzons et al. 2001; Jiménez-González et al. 2004b). The ACS GCI has also developed its own reagent and solvent selection guide publicly available, together with a solvent selection tool that provides a broad range of information about solvent properties to assist practitioners in choosing the best option (ACS 2023). The tool was originally developed by AstraZeneca plc and recreated by experts in pharmaceutical processes, and this special attention given confirms the great importance of organic solvents, as emphasized previously, due to their large impact and the variety of factors involved in the selection.

Many studies through the last 20 years suggest that the use of ionic liquids (ILs) could be revolutionary in that sense (Rogers and Seddon 2003; Zhao et al. 2005; Melo et al. 2013), being considered “green solvents” since they are non-volatile, stable, non-flammable, and having broad range of applicability (Choudhary et al. 2023; Yoo et al. 2017). However, a comparison between ILs and homologous organic solvents by means of a comprehensive life-cycle approach could give results different than expected (Zhang et al. 2008), since the “green” properties of ILs sometimes become negligible when considering the environmental impacts derived from the life cycle of the solvent itself (Chang 2020; de Jesus and Maciel Filho 2022; Maciel et al. 2019). For instance, a cradle-to-gate LCA study was conducted on the production of acetyl salicylic acid (ASA), comparing the process using toluene with the same pathway using [Bmim]Br (1-butyl-3-methylimidazolium bromide) as solvent, revealing that the production of ASA using the IL has dramatically higher life cycle impacts in all the nine categories considered (global warming potential, eutrophication potential, acidification potential, photochemical ozone creation potential, human toxicity potential, depletion of abiotic resources, aquatic ecotoxicity potential, terrestrial ecotoxicity, ozone layer depletion potential), especially due to the production of hydrogen bromide, butanol, methylamine, and glyoxal needed for the preparation of [Bmim]Br (Amado Alviz and Alvarez 2017). In the end, considering the whole life cycle, the production of ASA using toluene was the best option, considering the current recycling technologies of ILs as novel solvents (Sklavounos et al. 2016; Ren et al. 2021). This example indicates the strength of LCA methodology in assessing the environmental impact of chemical processes, demonstrating that a life-cycle approach is fundamental, and that any product, method, or technology should not be defined intrinsically “green,” but instead should be evaluated and contextualized in the relevant value chain. In the next sections, a focus on antibiotics is provided to support the discussion on the factors involved in the upstream phase and the approaches, methods, and tools that can be adopted in pharma-LCA.

#### Chemical synthesis: fluoroquinolones

In the case of upstream processing, the synthetic pathway chosen to prepare a chemical precursor can significantly affect the environmental impact of the overall system. A critical obstacle in pharma-LCA is the lack of inventory data about plant operations on a large scale (Jiménez-González et al. 2004a; Kralisch et al. 2015), which could be confidential and therefore not publicly available, as well as synthetic methodologies protected by industrial patents (Siegert et al. 2019b; Huber et al. 2022). In this context, LCA studies performed by pharmaceutical companies are beneficial to highlight the most impactful factors of the production (i.e., core phase), but they give only a partial picture of the actual environmental impact of the entire value chain. The upstream and downstream stages may be outside the physical boundaries of the company, but they could still influence the decision and management of the production by driving the purchase of the chemical precursors toward the most sustainable option(s) (Liou et al. 2021; Koenig et al. 2019).

For instance, Anastas and colleagues highlighted the lack of LCI data for large-scale production of pharmaceutical drugs and their precursors, performing a comparative cradle-to-gate analysis of different anesthetic APIs using a bottom-up approach (Parvatker et al. 2019). Starting from synthesis data at the lab-scale, these authors used chemical engineering methods for process design and scale-up to calculate the carbon footprint of 20 different anesthetic drugs, obtaining results ranging from 11 to 3000 kg CO_2_eq/kg of drug. However, the inventory data extrapolated from lab-scale experiments could produce emission values significantly overestimated, due to the large number of variables related to the scaling-up procedures (Piccinno et al. 2016; Simon et al. 2016; Thonemann and Schulte 2019; Elginoz et al. 2022). Another factor that needs to be emphasized is the choice between many different synthetic pathways available for the same drug, which was done arbitrarily by Parvatker et al. (2019), but it should be conducted according to the common materials and procedures applied in the region of interest. Despite these limitations, the LCI data obtained in this study could be used by LCA practitioners in future works, with the necessary adjustments needed by the case, offering a methodology to possibly develop similar inventories for other pharmaceutical drugs and chemical precursors. Moreover, it is worth highlighting that the results demonstrated poor correlations with the molecular weight and complexity of the substances studied, while the number of steps of the synthesis was a key parameter in determining the intensity of GHG emission released from the process.

When discussing the specific case of antibiotics, some important distinctions have been made, since these products are divided into several classes characterized by different mechanisms of action and chemical structures (Gould 2016). For instance, based on anatomical therapeutic chemical (ATC) therapeutic classes, a classification system used by the World Health Organization (WHO), antibacterial drugs fall in the macro category J (anti-infective for systemic use) and they are further divided into 10 sub-classes (WHO 2023). The chemical structures and production procedures differ from class to class, but a general distinction can be made between fully synthetic and biotechnological processes. In the case of synthetic antibiotics such as fluoroquinolones (J01MA), the synthesis of chemical precursors is generally very important and it should be included in cradle to grave LCA. The case of ciprofloxacin hydrochloride is commented below as an example of LCA application within the pharmaceutical sector (Yang et al. 2021), also conducted following the PCR document defined by Siegert et al. (2019a). PCR is a technical document that collects all the fundamental rules to perform a LCA study, in accordance with ISO 14040 and ISO 14044 (ISO 2006a; ISO 2006b), for the specific class of product they are developed for (in this case pharmaceuticals). PCRs are often used in type III label (ISO 2010) such as the Environmental Product Declaration (EPD) (Hunsager et al. 2014; Minkov et al. 2015; Ibáñez-Forés et al. 2016).

**Fig. 2:**
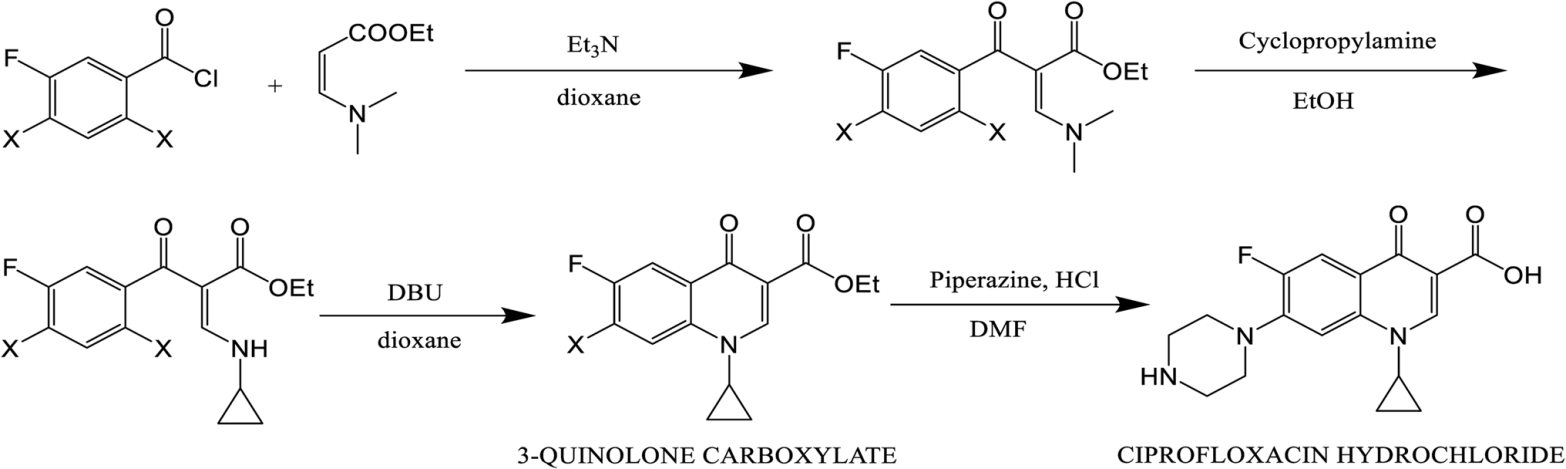
Traditional Grohe’s cycloaracylation route for the synthesis of fluoroquinolones. The procedure involves 1) benzoyl chloride condensation with an aminoacrylate derivative, 2) substitution of the N-terminal side chain, 3) cyclization obtaining the quinolone carboxylate structure, and 4) ester hydrolysis and piperazine addition

Ciprofloxacin hydrochloride is a fluoroquinolone antibiotic with a market size of 152.24 million USD in 2022 and an expected CAGR of 5.4% (Zion Market Research 2022). Yang et al. (2021) analyzed by LCA the core phase of the production of ciprofloxacin, and the results of the impact assessment stage showed that the API synthesis had the largest contribution to environmental impact (42.9%), followed by galenic formulation (41.9%), and packaging (15.2%). The higher impacts came from the energy consumption (mainly electricity from coal, since the study is located in China) and from the large amount of polyols used as solvent. The functional unit was the production of 280 million ciprofloxacin tablets and the system boundaries were set considering only the last step needed for the API synthesis starting from the quinolone carboxylate, which was not present in the ecoinvent database v.3.4 (Ecoinvent Centre 2021) and therefore placed under a relevant category (organic acid) to allow for software operation. Nevertheless, the synthesis of the quinolone involves at least three or four transformations with the common procedure, and even more if other pathways are used (Arava and Umareddy 2018). This means that considering all the processes needed to obtain the precursor the contribution to the environmental impact would be significantly higher; indeed other studies highlight the fact that the API synthesis generates more environmental impacts than galenic formulation and packaging (Jiménez-González et al. 2011b; Jung et al. 2021). For this reason, the optimization of chemical processes is recommended for reducing the overall footprint, and a great effort is put into finding new routes or optimizing the existing ones (Rose et al. 2022; Kar et al. 2022; Dunn 2012) as well as to track the improvements over the years. Ciprofloxacin was first synthesized by Bayer AG during the 1980s, thanks to Grohe’s cycloaracylation (Fig. 2), a multi-step method for the preparation of fluoroquinolones well described in a dedicated report by Bayer (2020). This route is still today the best option to produce ciprofloxacin and other quinolone antibiotics, e.g., enrofloxacin (Kong et al. 2021), while other routes developed by Natco Pharma Limited and Bayer AG present more steps for the synthesis and therefore are not convenient in economic terms and in terms of environmental impact (Muddasani and Nannapaneni 2001; Grohe et al. 1987).

Another procedure was developed by Suven Life Sciences Ltd. exploiting a Gould-Jacobs reaction to produce 3-quinolone carboxylate in one step but only afforded a 50:50 mixture of the desired product and a co-product (Arava and Bandatmakuru 2013). Later Bayer AG scientists managed to improve the original Grohe’s cycloaracylation starting from the same raw materials, designing a two-step process to significantly reduce the operations and chemicals needed (Zerbes et al. 1997). Following studies focused on the improvement of the selectivity of Grohe’s cycloaracylation, but these procedures involve the use of metals (FeCl_3_, Raney Ni, and CuCl) and the production of inorganic salts as waste, strongly increasing the environmental impact of the overall process (Muddasani and Nannapaneni 2001; Rao et al. 2012). More recent studies focused on the development of a continuous one-pot synthesis exploiting flow chemistry, with excellent results in terms of yield and greenness grade (Lin et al. 2017; Tosso et al. 2019). Moreover, another recent study described an efficient procedure for the late-stage addition of the piperazine derivative, exploiting a recyclable nano-zirconia catalyst in water, avoiding the use of organic solvents (Nakhaei et al. 2018).

**Fig. 3:**
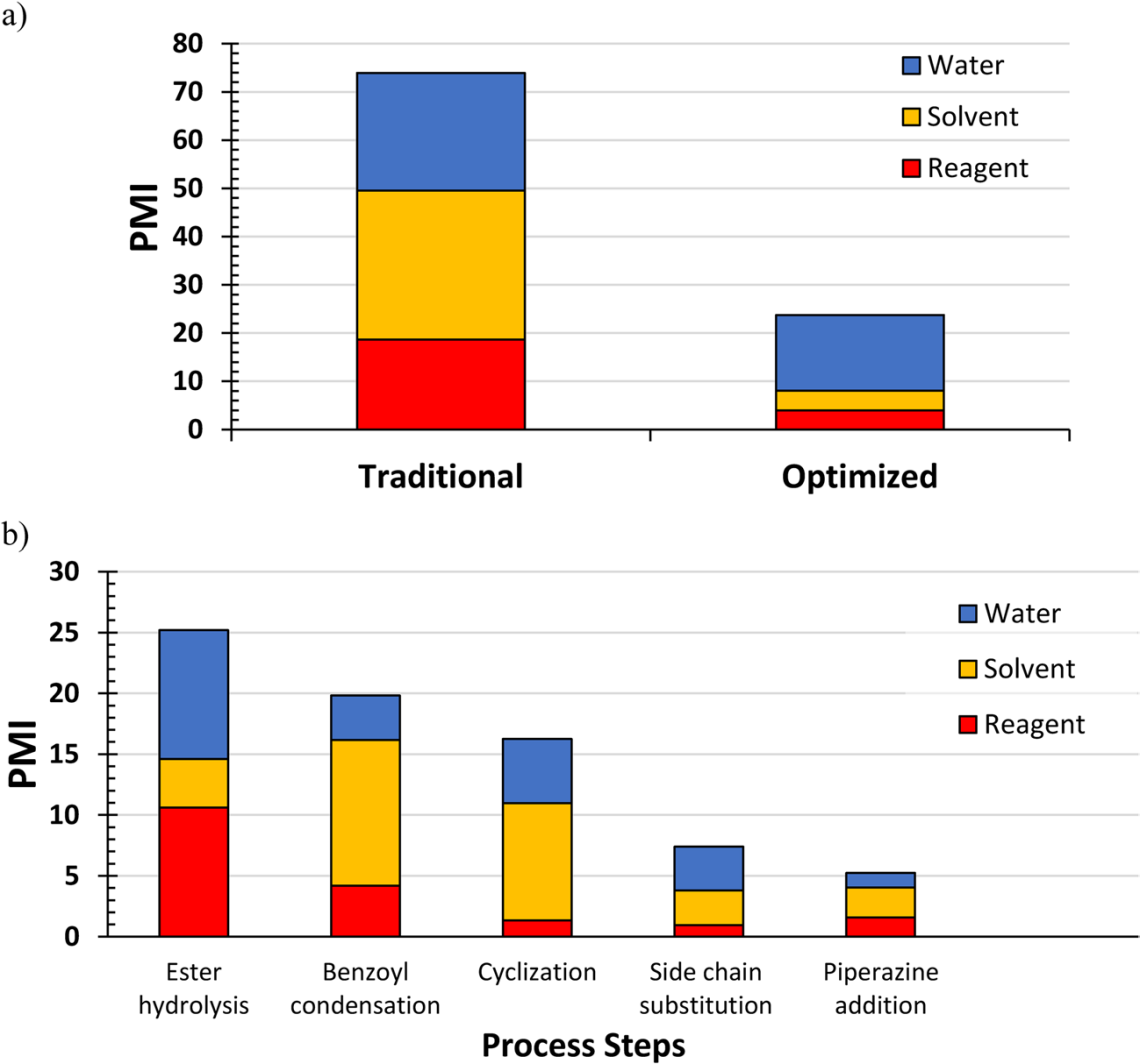
**a** Comparison of the PMI (process mass intensity) of the two processes, adapted from the results of the PMI-LCA tool; **b** PMI of each step of the traditional process, obtained using the PMI-LCA tool

**Fig. 4:**
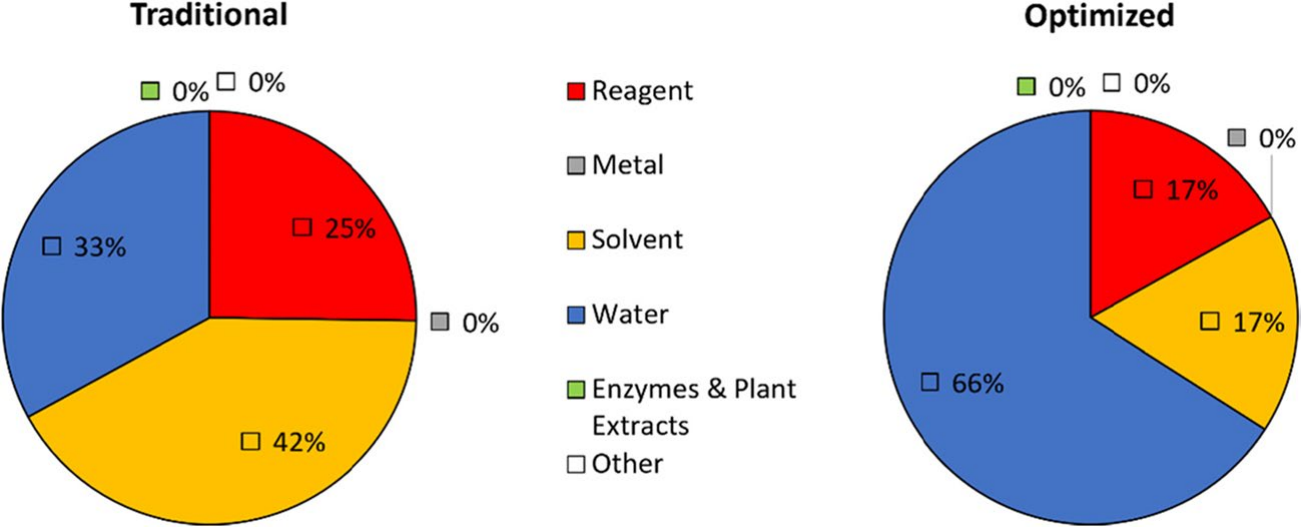
Breakdown of the PMI (process mass intensity) by class for the two processes, obtained using the PMI-LCA tool

**Fig. 5:**
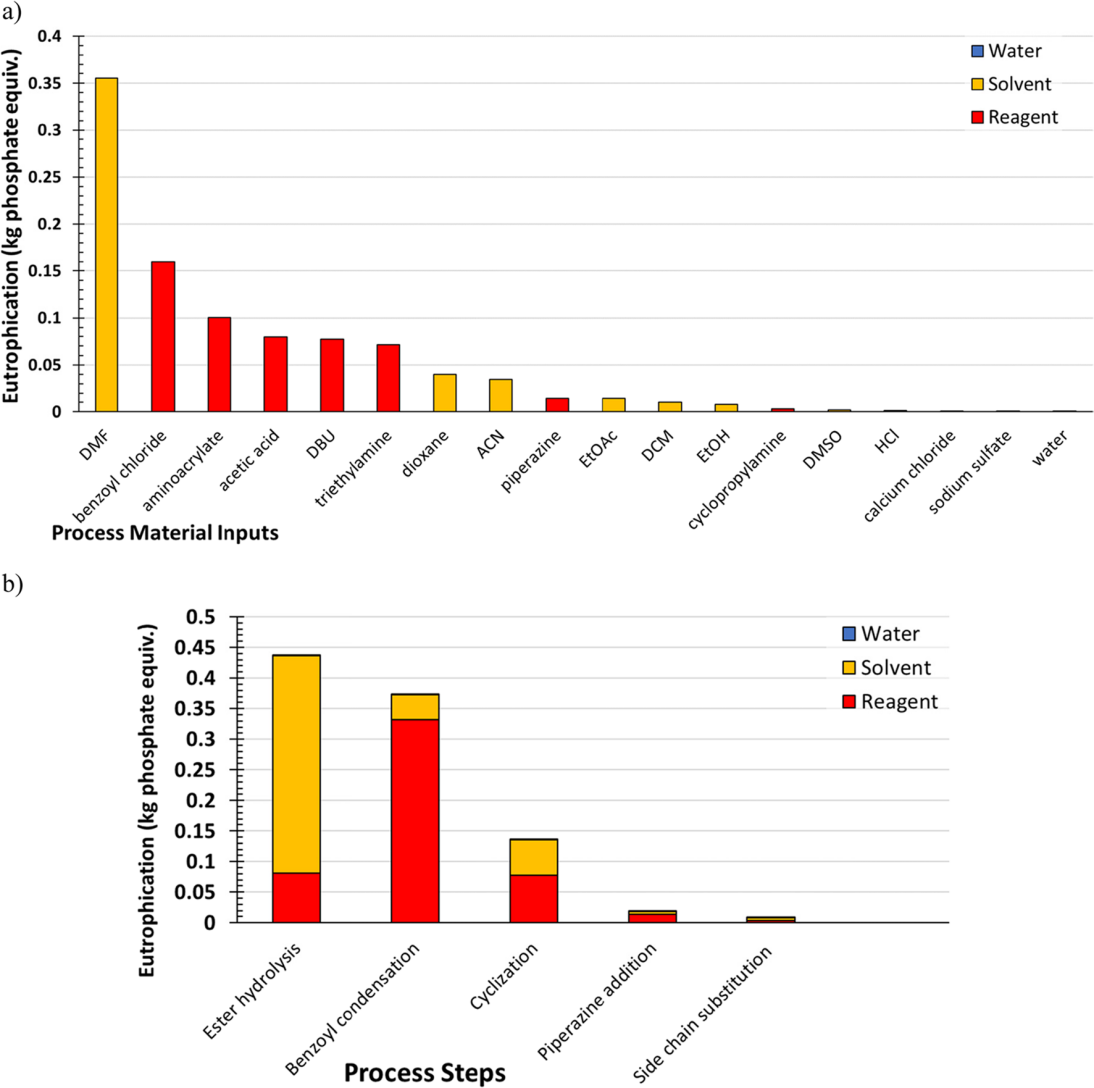
Contribution analysis to eutrophication potential of the traditional process, obtained using the PMI-LCA tool. **a** Materials; **b** steps

The PMI-LCA tool (ACS 2023), available from the ACS Green Chemistry Institute Pharmaceuticals Roundtable, provides a calculation of PMI based on experimental data and an estimation of life cycle information based on ecoinvent database, allowing to carry out a preliminary assessment of the environmental performance of processes for the synthesis of APIs, considering all the starting materials, solvents, reagents, catalysts, and other materials used. Employing the tool with experimental data from the referenced documents, it is possible to compare the results obtained by Bayer AG in the improvement of the cycloaracylation route, starting from the traditional method (Grohe and Heitger 1987) requiring five transformations and getting to the optimized two-step process (Zerbes et al. 1997). It should be noticed that the tool was planned for large-scale production of pharmaceuticals, indeed it provides PMI results in “kg of materials used/kg of packaged API produced,” but the packaging step is only a calculation made by the system to normalize steps on the production of 1 kg of API; therefore, the galenic formulation and the packaging stage are not considered in this assessment. Another consideration is that the two processes can be only evaluated separately, and after that the single results can be discussed. To make a reliable comparison, the scale of the two methods must be similar to avoid the intrinsic differences in the efficiency of procedures and technologies at different scales. In this case, both methods used approximately 30 g of benzoyl chloride and aminoacrylate (0.25 moles of each) as starting materials, enabling a consistent comparison and assessment of the two processes with the help of graphs and tables directly provided by the tool. As reported in Fig. 3a, which was adapted from the PMI results, it is revealed that a significantly higher impact is generated by the traditional process (Grohe and Heitger 1987) compared to the optimized two-step process (Zerbes et al. 1997), especially due to the higher amount of solvents (in yellow) and reagents (raw materials and reagents, in red) employed. The PMI was reduced from 74 to 24 kg/kg of API (− 68%), with the most important decrease detected in the quantity of organic solvents (almost 90% reduction) needed for the extraction and purification procedures. Figure 3b shows the most impactful steps of the traditional process in terms of material consumption, highlighting the great amount of solvents needed for the purification in benzoyl condensation and cyclization (steps 1 and 3) and the high amount of water needed for dilution of acids in the final ester hydrolysis. On the other hand, the PMI of the optimized process without considering water is just 8 kg/kg API, confirming the lower contribution derived from solvents (17%, in yellow) and reagents (raw materials and reagents, 17%, in red) as depicted in Fig. 4. After the calculation of the amounts of materials used (i.e., PMI) and the breakdown of these quantities, the tool provides also an estimation of the life cycle impacts of these materials, finally putting together all the resulting process metrics as reported in Tables 2 and 3. The graphs generated display the contribution of steps and materials to each of the six life cycle impact assessment (LCIA) categories (i.e., mass net, energy consumption, global warming potential, acidification potential, eutrophication potential, water depletion). Observing the results, the impact of the organic solvents in the traditional process is evident, especially due to the negative contribution of dioxane in the energy consumption, global warming potential (GWP), and water depletion categories, while DMF (dimethylformamide) is by far the biggest contributor to eutrophication. These impacts were revealed by contribution analyses made by the tool, highlighting the most impactful materials and steps for each of the six impact categories. For example, the contribution analysis for eutrophication potential of the traditional process is provided in Fig. 5. It can be appreciated the great contribution of DMF to the eutrophication potential of the overall synthetic route. The solvents used in the optimized process instead are toluene and NMP (N-methyl-2-pyrrolidone). Toluene is a better option compared to dioxane, while NMP appears as much hazardous as DMF; therefore, in more recent studies (Lin et al. 2017; Tosso et al. 2019) the choice has shifted to the more preferable dimethyl sulfoxide (DMSO). A great limitation is that the impacts derived from organic compounds used as reagents and raw materials could be underestimated, since they are processed by the software as “organic reagents” with the only distinction between bulk and fine chemicals (based on innovation green aspiration level, iGAL) (Roschangar et al. 2018). Still, this tool could be helpful to make a general analysis of a product life cycle that goes beyond a simple PMI calculation, and it could be further implemented with molecular structure-based distinctions for organic compounds.

**Table 2:** LCA results of optimized process, obtained using the PMI-LCA tool

Process metrics per kg API	Total	Reagent	Solvent	Water
PMI	23.77	4.01	4.08	15.67
Mass net (kg)	55.04	45.22	9.79	0.02
Energy (MJ)	1291.76	949.48	341.98	0.30
GWP (kg CO_2_ eq.)	101.26	86.56	14.68	0.02
Acidification (kg SO_2_ eq.)	0.74	0.70	0.05	0.00
Eutrophication (kg phosphate eq.)	0.27	0.10	0.17	0.00
Water (kg)	113.88	43.27	50.82	19.79

**Table 3:** LCA results of traditional process, obtained using the PMI-LCA tool

Process metrics per kg API	Total	Reagent	Solvent	Water
PMI	73.94	18.65	30.91	24.37
Mass net (kg)	292.05	233.05	58.96	0.04
Energy (MJ)	6993.44	4953.44	2039.54	0.46
GWP (kg CO_2_ equ.)	533.43	448.68	84.73	0.02
Acidification (kg SO_2_ eq.)	4.00	3.61	0.39	0.00
Eutrophication (kg phosphate eq.)	0.97	0.51	0.46	0.00
Water (kg)	575.59	229.98	314.81	30.79

The case of ciprofloxacin shows the importance of GC in improving the environmental performance of organic syntheses, with many other examples of API green pathways existing in literature (Kar et al. 2022; Dunn et al. 2010; Erythropel et al. 2018). The main motivations include i) the reduction of the synthetic steps, ii) the development of more converging routes, and iii) the minimization of the use of hazardous organic solvents such as dioxane, DMF, and dichloromethane (DCM). In the context of green synthesis of drugs, it could be also interesting to introduce the case of sildenafil citrate, which has been studied extensively (Dunn et al. 2004; Ouranidis et al. 2021) and comprehensively from cradle to API (Cespi et al. 2015). Scientists from Pfizer Inc. afforded a massive result in the process optimization from the medicinal chemistry route to the commercial process, leading to a reduction of impacts between 50 and 65% in terms of sustainability indicators such as PMI, cumulative energy demand (CED, MJ_eq_) (Frischknecht et al. 2007; Frischknecht et al. 2015), GWP (kg CO_2_eq/kg API), and human health endpoint indicator. Starting from simple PMI and E factor determination, a more comprehensive impact assessment was performed using ReCiPe 2008 (v1.11) (Goedkoop et al. 2013) at midpoint and endpoint indicators. As commented above in this work, the lack of inventory data (LCI) constitutes a serious drawback in the assessment of the upstream phase. In this study, the inventory was improved by exploiting the FineChem tool, a molecular structure-based model developed by Wernet et al. (2012), used to estimate key production and emission parameters starting from chemical structures. This kind of tools could be very useful to fill the data gap, together with data and correlations from research papers, public bodies, databases, and reports. In this perspective, a strong collaboration with pharmaceutical companies would be the key to performing a study aimed at including also the upstream stages.

#### Biotechnology: penicillins

Some classes of antibiotics like tetracyclines (ATC code J01AA), penicillins (J01C), and cephalosporins (J01D) are mainly produced by fermentation from bacteria or molds, and further chemically modified (Hook 2006). The LCI stage is different compared to synthetic antibiotics, due to the absence of intricate multi-step synthetic processes. Unfortunately, the low number of publications relevant to the application of LCA to pharmaceuticals (and fine chemicals in general) prepared through biotechnological processes makes the generation of the LCI a challenging task (Secchi et al. 2016), with the main inconsistencies and burden shifts encountered in the production of the feedstocks (Pietrzykowski et al. 2013; Renteria Gamiz et al. 2019). Usually, the raw materials are crop biomasses; therefore, their environmental performance strongly depends on the agriculture techniques and practices used as well as the water consumed and chemicals/nutrients applied to soil (i.e., NPK fertilizers and pesticides). The key substances typically used in the core phase of these processes are carbon sources and energy sources for the microbes (e.g., carbohydrates from renewable sources), a medium constituted of proteins and salt minerals, solvents for the extraction, and other auxiliaries if further modifications are necessary (Tufvesson et al. 2013; Moutousidi and Kookos 2021). For example, researchers at the Centre for Bioprocess Engineering Research (CeBER) of Cape Town University have performed a LCA of bio-synthetic penicillin V production exploiting penicillium fungi (Harding et al. 2018). These microorganisms normally produce common natural penicillin G (benzylpenicillin) but by providing a different lateral chain in the medium a similar drug can be obtained, with a different acyl-residue in the amino group in position 6. Following this strategy, bulky side chains are typically exploited to make the penicillin more resistant to hydrolysis by β-lactamase enzymes, obtaining a variety of compounds with better pharmacological properties and easier to administrate (Oshiro 1999). In the case of penicillin V, the lateral chain is obtained providing phenoxyacetic acid in the medium. This chemical is prepared from fossil resources, and it accounts in the LCI together with the reagents for the synthesis and its precursors (i.e., phenol, chloroacetic acid). However, the final LCIA of the study demonstrates that phenoxyacetic acid contribution to environmental impact is marginal, being between 5 and 10% in most of the impact categories (Harding et al. 2018). On the other hand, electricity, which is mainly provided from coal in the investigated system (i.e., South Africa), gives the biggest contribution to most of the impact categories, especially acidification (73%), eutrophication (37%), photochemical oxidation (72%), abiotic depletion (75%), and global warming (61%). Glucose production is the second contributor to acidification (12%), eutrophication (32%), and photochemical oxidation (7%), but it has also a negative contribution (− 9%) in global warming due to the CO_2_ uptake during the sugarcane growing. The contribution derived from glucose could be reduced using other carbon/energy sources from waste material (e.g., second-generation biomass), since the intensive monocultures of sugarcane replace natural habitat, leading to several issues such as decreased yield, soil acidification, and alterations in the microbial communities (Cespi et al. 2016).

Another strategy is the production of semi-synthetic penicillins, exploiting biocatalysis (Volpato et al. 2010). Immobilized enzymes (biocatalysts) are considered a very promising tool in green organic synthesis to avoid the use of metallic systems and the related hazards to the environment (Tao and Xu 2009; Sheldon and Woodley 2018). LCA studies demonstrate that biocatalytic routes have some key advantages compared to chemical catalysis, mainly the recyclability of the biocatalyst and the high specificity of enzymes (Henderson et al. 2008; Becker et al. 2023). Additionally, enzymatic reactions are typically performed in aqueous medium and under mild conditions, even if a considerable amount of organic solvents could be needed for extraction and purification (Delgove et al. 2019). Another drawback is that generally the production of immobilized enzymes could be energy intensive (Kim et al. 2009; Nielsen et al. 2007).

**Fig. 6:**
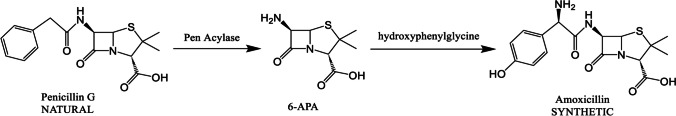
Preparation of semi-synthetic penicillins. The route consists of the production of natural penicillin G, then hydrolyzing the side chain obtaining the basic β-lactam structure 6-aminopenicillanic acid, which is then condensed with another sidechain

The enzyme *penicillin acylase* has been revolutionary in the field of antibiotic production, allowing chemists to prepare penicillins with various sidechains through a hydrolysis reaction that would be very challenging to do synthetically (Bruggink et al. 1998), and different mutants of the same enzyme are also capable of catalyzing the next reaction, the addition of the new sidechain (Xue et al. 2016). Through this strategy (Fig. 6), few synthetic chemicals and reagents are used, with a small contribution to the overall environmental impact of the supply chain. In the end, the production of penicillins has different issues regarding the upstream phase, and they are not strictly related to chemical synthesis. A possible drawback instead results in the downstream phase, since bio- and semi-synthetic penicillins are less sensitive to degradation by microbial enzymes and could be more persistent in the environment compared to natural ones (Xiao et al. 2021). This fact gains more importance considering that the occurrence of antibiotics (and APIs in general) in the environment is a serious concern for human health and for ecosystems, as stressed before in this review, and the assessment of this part of the life cycle of pharmaceuticals is discussed in the next section.

### Downstream processing

In the previous section, a description of the challenges related to the upstream phase has been commented, highlighting the use of GC principles in planning organic synthesis without going too deep into the LCA methodologies, setting the basis for a discussion about the sustainable production of APIs. The downstream phase, instead, presents different issues related to the fate and possible hazards derived from the release of drugs into the environment, focusing more on the actual pharma-LCA methodologies, the shortcomings, inconsistencies, and the main approaches emerging to overcome these challenges.

### Use and end-of-life phase of pharmaceuticals

In the case of pharmaceuticals, the use and EoL phase have a crucial impact on the LCIA, since the biological activity of the APIs produced could cause severe damage to the ecosystems if released into the environment. Moreover, the complexity and variety of the flows and emission pathways involved (Siegert et al. 2020) make the assessment of downstream processing a challenging task. These concerns become more severe considering the massive and continuous increase in the market of APIs worldwide. The research on the topic increased significantly in the last 20 years: the number of publications in the field of pharma-LCA during the 2011–2022 period was almost eight times higher than that of 2000–2011, describing a clear trend following the increasing interest in academic research in the subject of LCA application in the production and manufacturing sector, with the establishment of journals entirely devoted to the topic (Sabour et al. 2023).

Considering the existing pharma-LCAs, there are still many challenges to face in the methodology such as the data gap due to confidential information or the complexity of the supply chains, as commented above. These and similar limitations lead to a general lack of harmonization in the LCA studies for fine chemicals and more specifically for APIs, with the consequence of inadequate identification of the potential environmental impact of the life cycle of products. As we saw before, the main inconsistencies in the upstream phase regard the setting of the system boundaries when assessing chemical syntheses. For the downstream, instead, we will see that the quantification of API emissions in the environment is a crucial factor (Jiménez-González and Overcash 2014; Pålsson et al. 2019; Siegert et al. 2020). Several companies developed their own simplified tools to evaluate processes, for example, the SEEbalance® by BASF SE (Saling et al. 2002; Shonnard et al. 2003; Saling et al. 2005; Schmidt et al. 2004; Illner et al. 2014), but much information is not publicly available and there is still the need for general and universally accepted rules to perform LCA. Siegert et al. (2019a) developed some guidelines for drafting PCRs for pharma-LCA, setting some generic “horizontal” rules for pharmaceutical products and processes, which could be complemented by more specific “vertical” guidelines for each class of drugs such as antibiotics (J01), vasoprotectives (C05), or anesthetics (N01), based on the second level of ATC classification system (WHO 2023).

However, there are some disagreements about this categorization and the level of detail that these rules should reach, as highlighted by a survey taken by the European Commission’s Joint Research Center toward healthcare sector stakeholders, including industries, academic researchers, policy makers, and non-governmental organizations (de Soete et al. 2017). The majority of the participants in this survey claims that a product-specific approach is preferred to a group-oriented approach, mainly due to the differences in production processes and pharmacological effects of the different APIs. For example, all antibiotics could be framed within the same “vertical” PCRs or they could be separately considered based on, for instance, the third level of ATC classification, which distinguishes tetracyclines (J01AA), penicillins (J01C), and quinolones (J01M) into subcategories. Going further, the fourth level considers the chemical properties (e.g., fluoroquinolones J01MA), and finally the APIs are listed in the fifth level (e.g., ciprofloxacin J01MA02) (WHO 2023). Following the Guidance for Product Category Rule Development (GPCRD), Siegert et al. (2019a) proposed draft PCRs with the aim of stimulating the production of type III Environmental Product Declarations, based on established parameters according to ISO 14025 and ISO/TS 14027 (ISO 2010; ISO 2017). The EPD can be developed, in the end, for all types of products and groups with the aim of developing univocal LCA rules and methodologies to improve communication between producers, retailers, public administration, and customers, ultimately enabling consistent comparison between studies (Hunsager et al. 2014; Minkov et al. 2015; Ibáñez-Forés et al. 2016; Pålsson et al. 2019). The main issues to be discussed in drafting PCRs are the setting of the system boundaries, the definition of the functional unit (FU), and the assessment of the fate of products (EoL phase).

The mass-based FU (a.k.a. “declared unit,” kg of API or defined daily dose), commonly used to assess the upstream and the production processes, could be substituted by an effect-related FU, which considers the treatment of a patient with a certain disease for a certain period of time in a certain area (i.e., downstream). The need to expand the aim of the FU has been highlighted in different reviews and becomes more important in those LCA studies intended to assess the potential environmental impact of a product in the downstream phase of its life cycle, considering also eventual medical devices, auxiliaries, and drug delivery systems for the administration, in addition to the EoL stages (de Soete et al. 2017). As anticipated above, in the downstream stages (mainly use and EoL) lie the most significant gap in pharma-LCA, and the reason could be identified in the broad range of factors involved. Some of these factors could be quantified, such as the biodegradability of the API or its ecotoxicity. However, some other factors are very difficult to measure or even impossible without some kind of prediction or estimation related, for instance, to the amount of unused tablets and their effective disposal rate. An important contribution to the environmental impact comes from the possible ecotoxicological effects of APIs when released into the environment (Ortiz de García et al. 2017): municipal wastewater is pointed as the main source of these emissions, with particular attention placed on hospital effluents (Golbaz et al. 2021; Ulvi et al. 2022). Different public and private bodies are committed to provide ecotoxicological data and guidelines for environmental risk assessment of all the classes of chemicals (EPA in the USA, ECHA and CSTEE in EU, OECD aquatic toxicity classification), following the standard testing schemes and evaluation criteria of ISO standard specifications, for example, ISO 17088 and ISO 23517 (ISO 2021a; ISO 2021b). These data are then collected into ecotoxicological databases like ECOTOX (Olker et al. 2022) and characterized by predictive models such as USEtox® (Fantke et al. 2015) or the ecological structure activity relationships (ECOSAR, EPA 2023a) predictive model, providing tools for scientists to determine human and ecotoxicological impacts of chemicals and suggesting evidence for quantitative structure-activity relationships (QSAR, Sanderson et al. 2004).

However, to our knowledge, only a few existing studies fully considered the effects of drug emissions in their LCIA, and a comprehensive model for the evaluation of API flows in the downstream phase has not been properly developed yet. What is missing is the LCI data about the quantity of API that is likely released into the environment, especially from the wastewater (WW) effluent and sewage sludge (if used as fertilizer), which depends on the excretion and metabolization rates of APIs and of the different metabolites derived. This lack of information is generally related to an insufficient systematic monitoring of API occurrence in the WW treatment plant effluents and to the great variability of the available measured environmental concentrations (Arnold et al. 2014; Morais et al. 2014; Emara et al. 2019; Sanusi et al. 2023). Consequently, when carrying out an impact assessment, these variables should be modeled based on the geographical scope considering the disposal procedures, the local ecosystem, and the WWTP technology (Golbaz et al. 2021; Emara et al. 2019). A useful approach to estimate chemical emissions from WWTPs and exposure in surface water is the emission model SimpleTreat (v4.0), which estimates concentrations of contaminants in effluents and sludge, and the corresponding discharges through air (volatilization), solid, and liquid flows from the plant (Struijs 2014; Struijs 2015). SimpleTreat is a freely accessible software that has been exploited in a LCA study to assess emissions of amoxicillin, ciprofloxacin, and clarithromycin from WWTPs in Germany using an effect-related FU (Schulte et al. 2022). In this study a LCI previously developed by these authors was applied to estimate the flows of APIs in the use and EoL phase. First, research data and predictions from public sources are used to estimate the rates of administration, metabolization, excretion, and disposal of the pharmaceuticals. Second, quantitative determination of the three antibiotics in the WWTP inflow is carried out. Then, the SimpleTreat software enables to estimate the fate of the APIs, providing the rates of effluent discharge, biodegradation, and accumulation in sewage sludge based on a mass balance approach. With this additional information, the LCI model provides a general assessment of the flows and emission pathways of the APIs in the use and EoL phase. However, the model is still incomplete, especially regarding the antibiotic metabolites formed in the human body, and the final prediction strongly depends on the quality of input data.

In study of Schulte et al. (2022), the sources of the data about the physicochemical and environmental fate of the APIs (e.g., solubility and Henry constant) were extrapolated from research papers or predicted from EPI (Estimation Programs Interface) Suite™ (EPA 2023b), a software developed by US EPA. The absorption, excretion, and metabolization rates were taken from data sheets provided by pharmaceutical companies, and they are useful to estimate the rates of discharge of APIs into the influent. For pharma-related data like DDD, treatment period, or regular/irregular disposal rates, the source was the ATC index suggested by the WHO, providing the rates of administration. A loss rate of 5% was set by default due to the lack of comprehensive data, based on the EU Guidance for the Development of Product Environmental Footprint Category Rules (PEFCRs) published by the European Commission (2017), while the remaining part of the API administrated was assumed to be actually consumed by the patients. Finally, the SimpleTreat calculations estimated the fate of the three antibiotics entering the WWTP. The study shows how the ciprofloxacin is 50% discharged in the sewage treatment plant (STP in the figure) effluent, almost 25% accumulated in the sewage sludge, and a significative part (10–20%) assumed to be transformed into metabolites in the human body. In general, the limit of theoretical approaches like this one lies in the fact that different classes of pharmaceuticals might have different behaviors in terms of degradation and pharmacokinetic properties, ultimately affecting the model created.

Despite this, such an approach is appreciable to make a general estimation of API flows in the EoL phase but the actual emissions into the environmental compartments could be different since other processes occur after the WWTP effluent. Indeed, comparing the results of these estimations with empirical data is challenging due to the great variability of measured concentrations of contaminants in the environment. Thus, more investigations are needed to assess the fate of APIs after discharge, with further transformations and biological interactions happening in surface water, together with the implications of sludge treatment and its use as fertilizer. In the case of antibiotics these last considerations gain much more importance since the release of these APIs contributes to the enrichment of antimicrobial resistance (AMR) in microbial communities, which is discussed in the following section.

#### Antimicrobial resistance in life cycle assessment

As previously underlined, the case of antibiotics deserves special attention in LCA modeling, since the interest in their release in the environment is not only limited to toxicological effects in the ecosystems. Rather, there is an additional impact, that in the last decades has become a serious concern in the sector of pharmaceuticals and generally in the field of medicine: the enrichment of AMR (Polianciuc et al. 2020). Since the discovery of penicillin by A. Fleming, humanity faced a dramatic increase in well-being thanks to β-lactam antibiotics, opening possibilities for medical solutions to several diseases, and after the end of the Second World War antibiotics became widely available, with hundreds of new drugs developed worldwide (Gould 2016; WHO 2020; Cook and Wright 2022). In the recent times, antibiotics have been crucial for medicine, with more than 269 million antibiotic prescriptions in the USA only in 2015 (Centers for Disease Control and Prevention 2015). Same trend was observed in the EU: according to a 2019 census, an average consumption of antibiotics for systemic use (ATC group J01) was estimated to be 19.4 DDD per 1000 inhabitants per day (ECDC 2020). Therefore, the WHO defined AMR as one of the top 10 global public health threats facing humanity, since the misuse and overuse have favored the development of resistant populations through mutation, and their resistance-coding genes can be horizontally transferred and imported to other bacteria, generating issues in the treatment of certain diseases and consequently damage to human health, commonly measured in disability-adjusted life years (DALYs) (Jian et al. 2021). An important source of antibiotic emission are the wastewater effluents, like all the APIs (Kümmerer 2009), with most attention being placed on hospital wastewater monitoring (Yao et al. 2021; Canan-Rochenbach et al. 2023). Pharmaceutical’s production sites could also act as hotspots for AMR enrichment and as reservoirs for resistant genes due to emissions from effluents, and the lack of awareness about this contribution increases dramatically the urgency for standard methods and practices to mitigate the problem (Kotwani et al. 2021; Bombaywala et al. 2021).

Moreover, human consumption is not the only potential source, since globally the largest portion of antibiotics and antimycotics are used in animals for food production (van Boeckel et al. 2019), with dramatic consequences in the ecosystem, severely increasing the spreading of resistance-coding genes (Sanderson et al. 2004). Hence, the problem does not concern exclusively the application of LCA in the pharmaceutical industry, as the awareness on this topic is growing in all the sectors related to the use of antibiotics such as crop and livestock farming (Manyi-Loh et al. 2018) or aquaculture (Hossain et al. 2022), following the approach of One Health response for AMR emphasized by the WHO (2017). For instance, a recent study about rainbow trout production in the Spanish region of Galicia (Sanchez-Matos et al. 2023) included the AMR enrichment as a midpoint impact category in the LCIA to explore the consequences of antibiotics release in freshwater using USEtox®, and to date, this study is still the only reported in the literature that considers AMR in a life cycle evaluation.

As well known, USEtox® is a LCIA model based on scientific consensus developed by the UNEP and by the Society for Environmental Toxicology and Chemistry (SETAC) in the context of the life cycle initiative (Fantke et al. 2015). It provides midpoint and endpoint characterization factors for human toxicological (CF_hum_) and freshwater ecotoxicological (CF_eco_) impacts of chemical emissions in LCIA, to calculate the impact score (IS) based on the weighted summation of the potential contribution of the mass (*m*) of the emitted substance *x* released into compartment *i*.

1 $$IS=\sum_{x,i}{CF}_{x,i}{m}_{x,i}$$

**Fig. 7:**
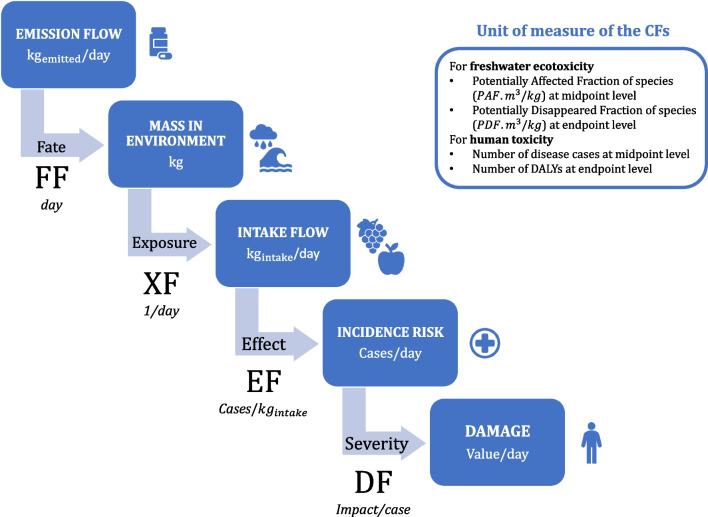
Framework for characterization of human toxicity and freshwater ecotoxicity in USEtox® 2.0 (Fantke et al. 2015)

CFs are derived from the product of three matrices including fate factors (FF), human exposure factors (XF), and human and ecotoxicological effect factors (EF). Then, to arrive at endpoint level, the midpoint CFs are multiplied by a severity factor, and the whole schematization is represented in Fig. 7 together with the units for the CFs at midpoint and endpoint level.

2 $$CF= FF\ast XF\ast EF$$

In their study, Sanchez-Matos et al. (2023) based the analysis on the model implemented by Nyberg et al. (2021). To our knowledge, this is the first attempt to include AMR in the pharma-LCA framework. The work carried out by Nyberg et al. (2021) presented a short review of the LCA-related studies in the literature dealing with antibiotics. These studies focus on freshwater ecotoxicity, calculating characterization factors (CF_eco_) with USEtox® or using available ones to conduct LCA of wastewater treatment. Nyberg et al. pointed out that the AMR phenomena are usually not considered or just mentioned in few of them. Then, starting from the USETox® framework, they proposed a viable strategy to characterize AMR enrichment by the definition of a new CF_AMR_ specifically designed for this purpose. They suggested two possible approaches: (A) characterizing AMR enrichment in the environment as a midpoint indicator and (B) characterizing impacts for AMR enrichment in human health endpoint indicator.

The first approach (A) is based on a methodology previously developed (Rico et al. 2017) for environmental risk assessment, in which minimum selective concentrations (MSCs) are inferred from minimum inhibitory concentrations (MICs). From MICs of antibiotics for pathogenic bacteria listed in the EUCAST database (European Committee on Antimicrobial Susceptibility Testing 2020), MSCs can be extrapolated, defined as the lowest concentration at which resistant strains manifest a competitive advantage toward analogous sensitives. It should be noticed that MSCs are lower than MICs, which means that resistance is promoted even at sublethal concentrations, and the authors defined by default an extrapolation factor of 10 (MSC/MIC ratio of 0.1). Then, the geometric mean of MSC data is used to extrapolate the HC_50_: the hazardous substance concentration that, at a given time, would promote AMR in 50% of species exposed. Similarly to what is done for the ecotoxicity, the effect factor for AMR enrichment (EF_AMR_) in environmental bacteria can be calculated by the USEtox® model as EF = 0.5/HC_50_, based on a linear extrapolation from the concentration-response curve and assuming that the acquisition of resistance at the community level increases with antibiotic concentration increase. The EF in ecotoxicity (EF_eco_) relates to the change in PAF (potential affected fraction of species) in response to an increase in contaminant concentration, while in the proposed model the EF_AMR_ refers to the change in the fraction of bacterial populations that acquire a significant increase in resistance after an increase in contaminant concentration. Finally, utilizing the established USEtox® framework based on Eq. (2), the authors introduced the new CF_AMR_ to characterize AMR enrichment in environmental bacteria as a midpoint indicator modifying only the EF. The MSCs are extrapolated from thousands of data about different bacteria found in the EUCAST database, rather than single dose-response experiments, assuming sufficient statistical robustness in the methodology. However, the extrapolation factor of 10 chosen to infer MSCs was based on a limited set of data about MSC/MIC ratios.

Emara et al. (2023) recently proposed a more comprehensive methodology to determine CFs for AMR, determining specific MSC/MIC ratios for each of the 2984 antibiotic-species combinations present in EUCAST database. This method used a more complex procedure involving a competition model that considers the fitness differences between sensitive and resistant strains (Greenfield et al. 2018). The MSC/MIC ratios estimated varied between 0.14 and 0.19 across the 2984 combinations included in the analysis, which means MICs are approximately five to eight times higher than MSCs, while Nyberg et al. (2021) assumed an extrapolation factor of 10 (ratio of 0.1) for all the antibiotics and species considered. Then, from these specific MSC data, Emara et al. (2023) derived for 128 antibiotics the specific RSC_5_ values (rather than HC_50_), defined as resistance selection concentrations that would promote AMR enrichment in 5% of exposed species. Finally, RSC_5_ data are used to calculate the EF and then the CFs for AMR enrichment in microbial communities. Moreover, these data could be used as a minimum threshold and compared to measured environmental concentrations in different compartments to determine the necessity of mitigation strategies. There are some limitations in this midpoint approach, related to the actual mutation dynamics, which depend strongly on the different levels and especially durations of contaminant exposure, rather than the concentration only. In fact, this approach seems to be better for monitoring small-scale systems with continuous emissions such as WWTP or other aquatic compartments, rather than more variable ones or regional scale systems. Moreover, in this approach, there is a lack of connection between the use of antibiotics and the possible human health impacts derived from resistance, due to the complex biological pathways and mechanisms involved.

In the second approach (B), proposed by Nyberg et al. (2021), this drawback is avoided by assuming that any use of antibiotics will contribute to resistance development in the bacteria community, enabling a characterization of potential impacts on a regional scale only based on the total use. The endpoint human health impacts of AMR are expressed as DALYs, the common unit used in LCA. The Joint Interagency Antimicrobial Consumption and Resistance Analysis (JIACRA) report (ECDC 2021) was used as data source, suggesting statistical correlations between consumption of antibiotics and AMR enrichment in human pathogens, together with other publications such as Cassini et al. (2019), reporting resistant pathogens and related impacts measured in DALYs. The model developed was based on the odds ratio (OR) from the JIACRA report, expressing a correlation coefficient *∂*_x,p,sect,reg_ explaining the relationship between the use in a certain sector and resistance development by a certain pathogen *p* toward a certain antibiotic *x* in the region of interest.

3 $${\partial}_{x,p,\mathrm{sect},\mathrm{reg}}=\sqrt{\ln \left({OR}_{x,p,\mathrm{sect},\mathrm{reg}}\right)}$$

Then, such a correlation coefficient was used together with EU data on antibiotic use (ABU), to express the total resistance by each pathogen (*ABF*_*x*, *p*, *reg*_) as the total (human + veterinary) resistance developed by a pathogen *p* toward a certain antibiotic *x* (Eq. 4). Finally, the *ABF*_*x*, *p*, reg_ is correlated to the data about impacts derived from resistant pathogens, and through a mass balance approach all the impacts from resistant pathogenic bacteria can be summed up, obtaining the DALYs per kg of antibiotic *x* used in a certain region of interest (Eq. 5).

4 $${\mathrm{ABF}}_{x,p,\mathrm{reg}}={\mathrm{ABU}}_{x,\mathrm{hum},\mathrm{reg}}\ast {\partial}_{x,p,\mathrm{hum},\mathrm{reg}}+{\mathrm{ABU}}_{x,\mathrm{vet},\mathrm{reg}}\ast {\partial}_{x,p,\mathrm{vet},\mathrm{reg}}$$

5 $$\frac{\mathrm{DALY}}{\mathrm{kg}\ {\mathrm{AB}}_x\ \mathrm{used}}=\frac{{\mathrm{DALY}}_{x,\mathrm{p}1,\mathrm{reg}}}{{\mathrm{AB}\mathrm{F}}_{x,\mathrm{p}1,\mathrm{reg}}} + \frac{{\mathrm{DALY}}_{x,\mathrm{p}2,\mathrm{reg}}}{{\mathrm{AB}\mathrm{F}}_{x,\mathrm{p}2,\mathrm{reg}}} + \dots +\frac{{\mathrm{DALY}}_{x,\mathrm{p}\mathrm{n},\mathrm{reg}}}{{\mathrm{AB}\mathrm{F}}_{x,\mathrm{p}\mathrm{n},\mathrm{reg}}\ }$$

An example of application is presented using the case of third-generation cephalosporins (3GC) in the EU, being crucial antibiotics for human medicine and making a big contribution to resistance-related mortalities, with 3GC-resistant *Escherichia coli* as the most critical pathogen. The OR from the JIACRA report and the annual data on antibiotics use from EU agencies (ABU, 270 tons for humans and 14 tons for veterinary) are used as input in the model, obtaining the ABF (resistance per kg per year). Then, with data on mortality caused by 3GC-resistant *E. coli* (37.2 DALYs per 100,000 population, corresponding to 191,883 DALYs across Europe the given population of 515.8 million in the year examined), Eq. (5) can be written in a simplified manner (Eq. 6), since it is considered only a class of antibiotics (3GC) and a pathogen (*E. coli*).

6 $$\frac{\mathrm{DALY}}{\mathrm{kg}\ 3\mathrm{GC}\ \mathrm{used}}=\frac{{\mathrm{DALY}}_{3\mathrm{GC},\mathrm{E}.\mathrm{coli},\mathrm{E}\mathrm{U}}}{{\mathrm{ABU}}_{3\mathrm{GC},\mathrm{hum},\mathrm{E}\mathrm{U}}\ast {\partial}_{3\mathrm{GC},\mathrm{E}.\mathrm{coli},\mathrm{hum},\mathrm{E}\mathrm{U}}+{\mathrm{ABU}}_{3\mathrm{GC},\mathrm{vet},\mathrm{E}\mathrm{U}}\ast {\partial}_{3\mathrm{GC},\mathrm{E}.\mathrm{coli},\mathrm{vet},\mathrm{E}\mathrm{U}}}$$

The final value of 0.87 DALY per kg of 3GC used is several orders of magnitude higher than values considering only direct toxicity impact (Ortiz de García et al. 2017), highlighting the importance of including AMR in the LCA of antibiotics. The limits of this approach are in the lack of data about AMR impacts, since the source used to develop this model (Cassini et al. 2019) only describes 16 pathogen-resistance combinations and the OR in the JIACRA report are limited as well. Moreover, the economic and nutritional losses from livestock mortality are not considered, together with the emissions of antibiotics derived from the core phase and from the transport.

Notwithstanding these limitations, the research described proposes two valuable approaches to deal with AMR, each one suitable for different time and spatial scales. The first approach seems to be more suitable for small-scale systems with continuous emissions (for example, a WWTP), but the connection with human health effects caused by resistant pathogens is lacking. In the second approach, instead, the endpoint human health impacts of AMR are expressed as DALYs, making it more suitable for system analysis at the regional scale, but it needs a strong background of data regarding AMR enrichment in human pathogens and related mortality rates in the region of interest. Starting from these concepts, many improvements could be made to make these models more accurate and adaptable to the different possible applications, considering the extensive use of antibiotics in various value chain of products. These improvements could lead to the development of more comprehensive and complementary environmental risk assessment methodologies. These approaches could be then commonly implemented as routine procedures when performing LCA studies of antibiotics, following the One Health framework stressed by the WHO (2017). This achievement, however, inevitably comes through an increase in the quality and quantity of monitoring data on antibiotic emissions, ultimately supporting a growing understanding of the relationships between the usage of antibiotics and resistant pathogens, as well as the complex dynamics of AMR enrichment in the microbial community. This knowledge could also provide the basis for quantitative estimation of threshold concentrations and define the urgency for remediation actions.

## Conclusion

In this short review, different challenges generally faced in pharma-LCA studies are discussed. We selected antibiotics as a case of discussion, given their importance in the field of pharmaceutical products. They represent among the classes of APIs more used in medicine and whose harmful effect on the environment is largely debated in literature. In the case of the upstream phase, the implementation of green metrics revolutionized the industry, driving the design of chemical processes toward more sustainable methods. However, comprehensive LCA studies are still necessary to quantify the environmental impacts of the whole supply chain of products. The lack of publicly available data related to large-scale operations for many chemicals sets serious limits to the implementation of LCA for pharmaceuticals. High-quality LCI data are crucial to assess the environmental impact of products considering the whole value chain “from cradle to grave.” About the downstream phases, the drawbacks related to the use and EoL stages derive from the complexity of flows and emission pathways involved. The approaches proposed to deal with these challenges suffer from the great variety of drug classes, each with different properties and interactions, hampering the development of a generalized framework for pharmaceuticals. The lack of systematic monitoring of drug emissions from WWTP effluents has been emphasized, together with a limited knowledge about the fate of human metabolites of APIs consumed.

In the field of pharma-LCA, we encourage the enhancement of collaboration and communication between producers, public administrations, and customers, defining common PCRs and supporting the development and adoption of universally accepted methodologies to minimize inconsistencies and facilitate the comparison of results. As underlined by WHO, the AMR enrichment is a global development threat, and a model to implement its consequences into pharma-LCA does not exist yet. Consequently, an important impact is excluded from the assessment of all the sectors related to the use of antibiotics. A recent study (Nyberg et al. 2021) proposed two different approaches to implement AMR as a midpoint or endpoint indicator, each one with different strengths and weaknesses that are discussed previously. The researchers have introduced interesting models with much space for improvement and discussion, and we suggest that it could be the starting point toward the development of a comprehensive methodology that is generally recognized and included in the LCA of products related with the use of antibiotics. The need of better knowledge regarding the causes and consequences of AMR enrichment has been stressed extensively, together with a more general lack of available data regarding emissions of antibiotics in the downstream phase.

Finally, it should be noted that these linear dose-response models do not consider the beneficial aspects of antibiotics use, being a pillar in modern medicine with millions of lives saved and many hospitalizations avoided each year, making the sustainability of antibiotics (and this is true in general for pharmaceuticals) a very complex topic that needs much more discussion to find a common consensus and the inclusion of further analysis to address all the social benefits (e.g., social-LCA).
